# Comparison of Metronidazole Gel and Povidone‑Iodine for Vaginal Preparation in Preventing Post‑Cesarean Infection‑Related Morbidity: A Three‑Group Randomized Controlled Trial

**DOI:** 10.1002/hsr2.73395

**Published:** 2026-10-14

**Authors:** Fatemeh Davodabadi, Hamideh Parsapour, Behzad Imani, Salman Khazaei, Mahnaz Moghadari Koosha

**Affiliations:** ^1^ Student Research Committee, School of Allied Medical Sciences Hamadan University of Medical Sciences Hamadan Iran; ^2^ Assistant Professor of Perinatology, Clinical Research Development Unit of Fatemieh Hospital, Department of Obstetrics and Gynecology, School of Medicine Hamadan University of Medical Sciences Hamadan Iran; ^3^ Department of Operating Room, School of Allied Medical Sciences Hamadan University of Medical Sciences Hamadan Iran; ^4^ Research Center for Health Sciences Hamadan University of Medical Sciences Hamadan Iran

**Keywords:** cesarean, endometritis, infectious morbidity, metronidazole, povidone‐iodine, vaginal preparation

## Abstract

**Background and Aims:**

Despite routine prophylactic antibiotics, post‐cesarean infectious morbidity remains a major challenge. Preoperative vaginal preparation can reduce vaginal microbial load, but direct comparisons are scarce. This study compared metronidazole gel versus povidone‐iodine for preventing post‐cesarean infection‑related complications.

**Methods:**

This three‑arm randomized controlled trial was conducted at Fatemieh Hospital, Hamadan, Iran, from December 2025 to May 2026. A total of 201 women scheduled for elective cesarean delivery were randomized to metronidazole gel 0.75%, povidone‐iodine 10%, and control (*n* = 67 each). Primary outcomes were endometritis, postoperative fever, and surgical site infection (SSI). Secondary outcomes included need for additional antibiotics, length of hospital stay, readmission, and adverse events. Data were analyzed in Stata v.17 using ANOVA and chi‐square tests (*p* < 0.05).

**Results:**

The composite infectious morbidity was significantly lower in the metronidazole group (6/67, 8.96%) compared to controls (21/67, 31.34%) (risk difference: 22.4%; 95% CI: 9.5%–35.3%; *p* = 0.002). Endometritis rates were 1/67 (1.49%), 3/67 (4.48%), and 8/67 (11.94%) in the metronidazole, povidone‐iodine, and control groups, respectively (*p* = 0.03). Fever rates were 2/67 (2.99%), 5/67 (7.46%), and 10/67 (14.93%) (*p* = 0.04). Need for additional antibiotics was lower in the metronidazole group (7/67, 10.45%) versus controls (18/67, 26.87%) (*p* = 0.04). Only the composite outcome remained significant after Bonferroni correction; individual outcomes did not. No significant differences were observed for SSI, readmission, or length of stay.

**Conclusions:**

Metronidazole gel significantly reduced composite infectious morbidity compared with no vaginal preparation, an effect that remained significant after adjustment for multiple comparisons. While individual outcomes showed clinically meaningful reductions, statistical significance was not maintained after Bonferroni correction. Metronidazole gel may be a promising option for vaginal preparation, particularly in high‐risk settings.

**Trial Registration:**

IRCT20251227068466N1.

## Summary

- What is already known: Preoperative vaginal preparation may reduce infection‑related complications after cesarean delivery, but direct comparisons between metronidazole gel and povidone‐iodine are limited.
- What is new: This randomized controlled trial demonstrates that metronidazole gel significantly reduces composite infectious morbidity compared with both povidone‐iodine and no vaginal preparation.
- Clinical implications: Metronidazole gel may represent a safe and practical vaginal preparation strategy before cesarean delivery that may reduce healthcare costs by decreasing the need for additional antibiotics, particularly in high‐risk settings.

## 1 Introduction

Cesarean delivery is one of the most common and rapidly growing major surgical procedures in women worldwide, with its rates exceeding 20% of all deliveries [1]. This increase, combined with the invasiveness of surgery, has made post‐cesarean infections a major maternal health challenge. Endometritis, surgical site infection, sepsis, and postoperative fever are among the most common complications, imposing a considerable economic burden on patients and the healthcare system [2, 3]. Although prophylactic systemic antibiotics reduce infection by up to 60% [4], the incidence of these complications remains 5–20 times higher than that of vaginal delivery [5], indicating the inadequacy of this intervention alone.

Most pelvic infections after cesarean section are of endogenous origin, as microorganisms from the lower genital tract ascend to the abdominal cavity during cervical manipulation and instrument passage and reach the sterile uterine environment and incision site [6, 7]. Therefore, reducing the preoperative vaginal microbial load is considered a logical complementary strategy. Numerous studies, including the Cochrane systematic review, have confirmed the effectiveness of vaginal preparation in reducing infectious complications [8].

Among different vaginal preparation methods, povidone‐iodine solution and metronidazole gel represent two distinct pharmacological approaches: povidone‐iodine is a broad‐spectrum antiseptic with rapid, non‑specific action that eliminates a wide range of microorganisms [8]. Due to its low cost, availability, and endorsement by the World Health Organization, it is the most common vaginal antiseptic in many countries [9]. In contrast, metronidazole is a targeted antimicrobial agent that specifically acts against gram‑positive and gram‑negative anaerobic bacteria—organisms that play a key role in the pathogenesis of post‑cesarean endometritis [10, 11]. However, because of the limited number of studies on metronidazole, it is not widely used in clinical practice [12], and a direct comparison between these two agents was not found in our literature search.

Furthermore, published network meta‑analyses have each identified different agents as the most effective option for preventing post‑cesarean infection. This heterogeneity among the strongest available evidence indicates a lack of final consensus on the best agent, and this disagreement mainly stems from the scarcity of direct comparative studies between different approaches. In this context, metronidazole remains a potential but severely under‑investigated alternativ [12, 13]. Consequently, it remains unclear which of these two approaches, the broad‑spectrum antiseptic or the specific anaerobic antibiotic, is more effective in reducing post‑cesarean infectious complications. This research gap is especially important in teaching hospitals with high delivery volumes.

Accordingly, the present study was designed and conducted to compare the effect of preoperative vaginal preparation with metronidazole gel 0.75% versus povidone‐iodine 10% on primary and secondary infectious outcomes after cesarean delivery. Our hypothesis was that, given the predominant role of anaerobic bacteria in the pathogenesis of these infections, targeted vaginal preparation with metronidazole may be clinically more effective than povidone‐iodine. The results of this study can provide more precise evidence for determining the optimal method of infection prevention after cesarean delivery.

## 2 Materials and Methods

### 2.1 Study Design and Setting

This study is a randomized, parallel‐group, three‐arm controlled trial. This trial was registered with the Iranian Registry of Clinical Trials (IRCT) on December 29, 2025, under registration number IRCT20251227068466N1. Participant enrollment commenced on December 30, 2025, after the trial registration was completed, and continued until May 30, 2026. The study was conducted at Fatemieh Educational and Treatment Center in Hamadan, Iran.

### 2.2 Randomization and Blinding

Eligible participants were assigned using permuted block randomization with a block size of 6 in a 1:1:1 ratio to one of three arms(1): vaginal preparation with metronidazole gel 0.75%, (2), vaginal preparation with povidone‑iodine (Betadine) solution 10%, and (3) control group (standard care without vaginal preparation). To ensure allocation concealment, the randomization sequence was generated by an independent statistician external to the clinical team and placed in sequentially numbered, opaque, sealed envelopes. After definitive enrollment and immediately before the start of surgery, the corresponding envelope was opened and the assigned intervention was determined.

Participants and the statistical analyst were blinded to the group assignments. To maintain blinding of participants, cloth drapes were placed over the upper abdomen during preoperative preparation so that the vaginal preparation (or its absence) could not be seen by the patient. The intervention providers were not blinded due to the different physical nature of the interventions (gel application, liquid irrigation, or no preparation). The group allocation codes were only disclosed (unblinded) after the final statistical analysis had been completed. The trial was reported in accordance with the CONSORT 2025 Statement [14].

### 2.3 Sample Size Calculation

Based on the study by Agbana et al. (2024), assuming an endometritis rate of 25% in the control group (P1 = 0.25) and 3.5% in the intervention group (P2 = 0.035), with a power of 80% (*β* = 0.20) and a type I error of 0.05 (*α* = 0.05), the minimum required sample size was calculated as 50 participants per arm [15]. Considering a predicted dropout rate of 25% (due to the long 42‐day follow‐up period and the challenges of postpartum follow‐up), the final sample size was increased to 67 participants per arm.

$n=\frac{{\left(Z_{1-\alpha /2}+Z_{1-\beta }\right)}^{2}[P_{1}\left(1-P_{1}\right)+P_{2}(1-P_{2})]}{{\left(P_{1}-P_{2}\right)}^{2}}.$

### 2.4 Inclusion and Exclusion Criteria

#### 2.4.1 Inclusion Criteria

- Maternal age between 18 and 45 years.
- Singleton, term pregnancy (37–42 completed weeks of gestation).
- Candidate for elective cesarean delivery.
- Provision of written informed consent to participate in the study.

#### 2.4.2 Exclusion Criteria

- Premature rupture of membranes (PROM) before the start of surgery.
- Chorioamnionitis or any sign of active systemic infection at admission
- Fever (body temperature ≥ 38°C) at admission.
- Pathological and abnormal vaginal discharge (accompanied by foul odor, burning, or severe pruritus).
- History of systemic antibiotic use within the 30 days preceding surgery.
- Immunodeficiency or insulin‐dependent diabetes mellitus.
- Known allergy or hypersensitivity to iodine or metronidazole.
- Maternal unwillingness to continue participation at any stage of the study.

### 2.5 Interventions

To standardize the process, all participants in the three groups received a prophylactic dose of systemic antibiotics (2 g intravenous cefazolin) approximately 30 min before skin incision [16]. All surgical procedures were performed by the same surgical team using spinal anesthesia according to an identical protocol. Before incision, abdominal skin preparation with 10% povidone‐iodine solution was carried out in all three groups following the national standard protocol. The specific protocol for each study arm (using a separate sterile glove, before abdominal skin preparation) was applied as described below.

#### 2.5.1 Group 1: 0.75% Metronidazole Gel

In this group, 5 g of 0.75% metronidazole gel (one full applicator) was placed intravaginally in close contact with the fornices and cervix under completely aseptic conditions. After expelling the gel, the applicator was carefully removed, and the vagina was not washed or wiped until the end of the surgical procedure to allow maximal local drug absorption.

#### 2.5.2 Group 2: 10% Povidone‐Iodine (Betadine)

In this arm, sterile gauze soaked in 10% povidone‐iodine solution was applied using sterile forceps with 360° rotating movements for 30–60 s from the upper vagina (cervix and fornices) toward the vaginal introitus, so that all anterior, posterior, and lateral vaginal walls were thoroughly coated with the solution; the gauze was then removed.

#### 2.5.3 Group 3: Control (No Vaginal Preparation)

Patients in this group received no vaginal preparation or washing before cesarean delivery and were managed only with routine ward care (prophylactic antibiotics and abdominal skin preparation).

### 2.6 Outcome Definitions and Assessment

All participants were followed for 42 days after cesarean delivery for the occurrence of infectious complications and outcomes. Clinical assessments were performed on postoperative days 3, 10, and 42 by a trained research nurse who was not blinded to group allocation. However, to minimize potential detection bias, all suspected cases of infectious complications were independently reviewed and confirmed by an attending obstetrician‐gynecologist who was blinded to treatment allocation. Outcome data were recorded using standardized case report forms, and predefined diagnostic checklists based on the Centers for Disease Control and Prevention (CDC) and National Healthcare Safety Network (NHSN) criteria were used for outcome assessment.

#### 2.6.1 Primary Outcomes

- Endometritis: clinical diagnosis based on the presence of at least two of the following signs in the absence of other sources of infection: fever (oral or axillary temperature ≥ 38°C), uterine tenderness on palpation, or purulent, foul‐smelling vaginal discharge (infected lochia) [17].
- Postoperative fever: body temperature ≥ 38°C on two separate occasions at least 6 h apart, after the first 24 h following surgery and up to Day 10 [18].
- Surgical site infection (SSI): presence of purulent drainage from the incision or spontaneous wound dehiscence accompanied by at least one inflammatory sign including erythema, warmth, local swelling, or severe pain [19].
- Composite infectious complications: a composite outcome defined as the occurrence of at least one of the three complications above (endometritis, postoperative fever, or SSI).

#### 2.6.2 Secondary Outcomes

- Need for additional antibiotics beyond the initial prophylactic dose.
- Length of hospital stay (days).
- Rate of unscheduled outpatient or emergency readmission due to postpartum infectious symptoms within 42 days.
- Occurrence of any intervention‑related adverse events, including local burning sensation, pruritus, mucosal irritation, skin rash, allergic reactions, or significant vaginal discomfort requiring medical attention.

### 2.7 Statistical Analysis

Data were analyzed using Stata version 17.0. The normality of continuous variables was assessed using the Shapiro–Wilk test, and homogeneity of variances was evaluated with Levene's test. Normally distributed continuous variables are presented as mean ± standard deviation (SD) and compared using one‐way ANOVA. Categorical variables (including baseline characteristics and outcomes) are presented as frequencies (percentages) and analyzed using the chi‐square test or Fisher's exact test, as appropriate.

When the chi‐square test was significant, pairwise post‐hoc comparisons were performed using Bonferroni correction, with an adjusted significance level of 0.0167 (0.05/3).

The composite infectious morbidity outcome was designated as the primary endpoint. Endometritis, postoperative fever, and surgical site infection (SSI) were prespecified components of this composite outcome. No formal hierarchical testing strategy or prespecified multiplicity adjustment was applied to these individual components; therefore, their analyses should be interpreted as supportive and exploratory. The need for additional antibiotics, length of hospital stay, readmission, and intervention‐related adverse events were prespecified secondary outcomes. No subgroup analyses were prespecified or performed.

To adjust for potential confounders, a multivariable logistic regression model was performed for the composite outcome, including intervention group, age, BMI, gestational age, preoperative hemoglobin, gravidity, parity, and education level as covariates. Results are presented as adjusted odds ratios (OR) with 95% confidence intervals (CIs).

All statistical tests were two‐sided. The a priori significance level was set at *p* < 0.05 for the primary overall analyses. For Bonferroni‐adjusted pairwise comparisons, statistical significance was defined as *p* < 0.0167.

## 3 Results

### 3.1 Baseline Characteristics

A total of 201 participants were enrolled according to the inclusion criteria (67 patients per group). All patients completed the 6‐week follow‐up period, and no dropout occurred during the study (Figure 1). As shown in Table 1, there were no significant differences between the three groups regarding age, body mass index, gestational week, preoperative and postoperative hemoglobin, gravidity, parity, or level of education (*p* > 0.05). Additionally, the standardized mean differences (SMD) for all continuous variables were below 0.3, with the majority below 0.2, indicating adequate balance between the groups.

**Figure 1:**
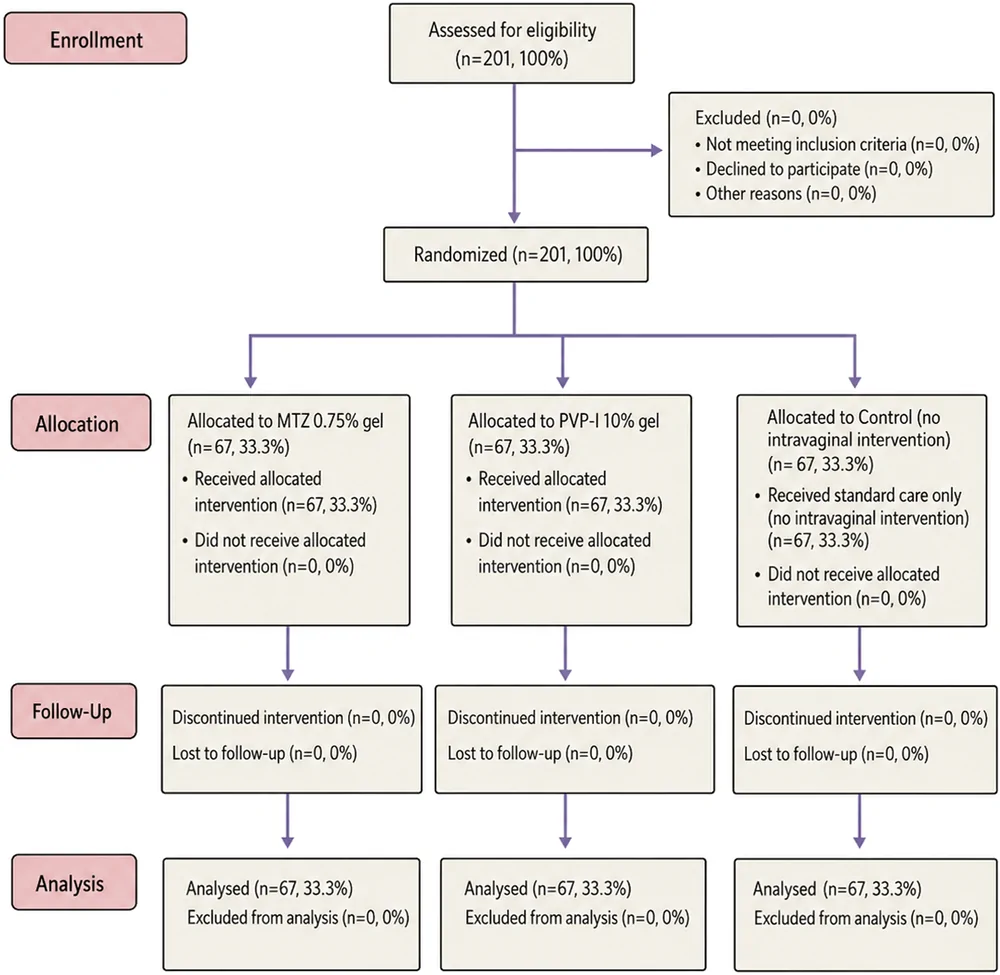
CONSORT 2025 Flow Diagram for a Three‐Group parallel randomized controlled trial. This diagram shows the flow of participants through each stage of the trial. *n* = number of participants. Percentages are calculated based on the number assessed for eligibility (*N* = 201), MTZ = metronidazole 0.75% vaginal gel; PVP‐I = povidone‐iodine 10% vaginal gel; Control = no intravaginal intervention.

**Table 1:** Comparison of baseline demographic and clinical characteristics between three groups.

Characteristics		povidone‐iodine (*n* = 67)	Metronidazole gel (*n* = 67)	Control (*n* = 67)	*p* value*	SMD^†^
Age (yr), mean (SD)		31.75 (5.95)	31.60 (6.26)	31.99 (5.43)	0.93	0.07
BMI (kg/m^2^), mean (SD)		30.78 (4.32)	29.98 (3.87)	30.43 (3.65)	0.50	0.20
Gestational age (wk), mean (SD)		37.88 (0.56)	37.96 (0.53)	37.81 (0.56)	0.30	0.28
Preoperative Hb (g/dL), mean (SD)		11.70 (0.91)	11.95 (0.79)	11.76 (0.84)	0.20	0.29
Postoperative Hb (g/dL), mean (SD)		10.58 (0.52)	10.62 (0.67)	10.58 (0.63)	0.92	0.07
Gravidity, mean (SD)		2.8 (1.1)	2.7 (1.2)	2.6 (1.0)	0.45	0.23
Parity, mean (SD)		2.5 (0.9)	2.4 (1.0)	2.4 (0.8)	0.68	0.21
Level of education, No. (%)	Primary education	32 (47.76%)	32 (47.76%)	34 (50.75%)	.44	—
	Secondary education	30 (44.78%)	26 (38.81%)	30 (44.78%)		
	Tertiary education	5 (7.46%)	9 (13.43%)	3 (4.48%)		

*Note:* Statistical significance was set at *p* < 0.05. *p value from one‐way ANOVA (for continuous variables) and chi‐square or Fisher's exact test (for categorical variables). †Standardized mean difference (SMD); values represent the maximum absolute pairwise SMD between any two groups for each variable.

Abbreviations: BMI, Body Mass Index; Hb, hemoglobin; SD, standard deviation.

### 3.2 Primary Outcomes

#### 3.2.1 Endometritis

The incidence of endometritis was 3/67 (4.48%) in the povidone‐iodine group, 1/67 (1.49%) in the metronidazole gel group, and 8/67 (11.94%) in the control group (chi‐square test, *p* = 0.03) (Table 2). In the pairwise comparison between the control and metronidazole groups, the risk difference was 10.4% (95% CI: 2.2%–18.7%; *p* = 0.016). However, after Bonferroni correction, the adjusted *p* value was 0.05, which exceeded the corrected significance threshold of 0.0167 and was therefore not considered statistically significant (Table 3). No significant differences were observed between the povidone‐iodine and control groups or between the povidone‐iodine and metronidazole gel groups after Bonferroni correction (Table 3). The overall difference among the three groups was significant (*p* = 0.032) (Table 2).

**Table 2:** Comparison of primary and secondary outcomes between the three study groups.

Outcome		Povidone‐iodine (*n* = 67)	Metronidazole gel (*n* = 67)	Control (*n* = 67)	*p* value*
Primary outcomes	Endometritis, No. (%)	3 (4.48)	1 (1.49)	8 (11.94)	0.03
	fever, No. (%)	5 (7.46)	2 (2.99)	10 (14.93)	0.04
	Surgical site infection (SSI), No. (%)	7 (10.45)	4 (5.97)	11 (16.42)	0.15
	Composite outcome, No. (%)	10 (14.93)	6 (8.96)	21 (31.34)	0.002
Secondary outcomes	Need for additional antibiotics, No. (%)	11 (16.42)	7 (10.45)	18 (26.87)	0.04
	Readmission, No. (%)	16 (23.88)	14 (20.90)	19 (28.36)	0.60
	Length of hospital stay (> 1 day), No. (%)	1 (1.49)	1 (1.49)	2 (2.99)	0.78

*Note:* Statistical significance was set at *p* < 0.05. *p value from chi‐square test for overall comparison among the three groups.

**Table 3:** Pairwise comparisons of primary and secondary outcomes with Bonferroni correction.

Outcome		Comparison	Difference (95% CI)	RR (95% CI)	*p* value*	*p* value (Bonferroni ‐corrected)^†^
Primary outcomes	Endometritis	Metronidazole versus Control	−10.45 (−18.74 to −2.16)	0.12 (0.02 to 0.97)	0.02	0.05
		Povidone‐iodine versus Control	−7.46 (−16.67 to 1.75)	0.38 (0.10 to 1.35)	0.12	0.35
		Metronidazole versus Povidone‐iodine	−3.00 (−8.70 to 2.80)	0.33 (0.04 to 3.12)	0.31	0.93
	fever	Metronidazole versus Control	−11.94 (−21.40 to −2.48)	0.20 (0.05 to 0.88)	0.02	0.05
		Povidone‐iodine versus Control	−7.46 (−18.06 to 3.14)	0.50 (0.18 to 1.38)	0.16	0.47
		Metronidazole vs Povidone‐iodine	−4.50 (−11.90 to 2.90)	0.40 (0.08 to 1.99)	0.25	0.74
	Composite infectious complications	Metronidazole versus Control	−22.39 (−35.43 to −9.34)	0.29 (0.12 to 0.66)	0.00	0.00
		Povidone‐iodine versus Control	−16.42 (−30.42 to −2.41)	0.48 (0.24 to 0.93)	0.02	0.07
		Metronidazole versus Povidone‐iodine	−6.00 (−16.90 to 4.90)	0.60 (0.23 to 1.56)	0.29	0.86
Secondary outcomes	Need for additional antibiotics	Metronidazole versus Control	−16.42 (−29.31 to −3.52)	0.39 (0.17 to 0.87)	0.02	0.05
		Povidone‐iodine versus Control	−10.45 (−24.28 to 3.38)	0.61 (0.31 to 1.19)	0.14	0.43
		Metronidazole versus Povidone‐iodine	−6.00 (−17.50 to 5.50)	0.64 (0.26 to 1.54)	0.31	0.93

*Note:* Pairwise comparisons were not performed for outcomes with a non‐significant overall chi‐square test (SSI, *p* = 0.15; readmission, *p* = 0.60; length of hospital stay, *p* = 0.78). *p value from the chi‐square test for each pairwise comparison without adjustment. †Bonferroni‐adjusted significance threshold: *p* < 0.0167 (0.05/3) for the three pairwise comparisons within each outcome.

Abbreviations: CI, confidence interval; Diff, absolute percentage difference between proportions; RR, risk ratio.

#### 3.2.2 Postoperative Fever

The incidence of fever was 5/67 (7.46%) in the povidone‐iodine group, 2/67 (2.99%) in the metronidazole gel group, and 10/67 (14.93%) in the control group (chi‐square test, *p* = 0.04) (Table 2). In the pairwise comparison between the control and metronidazole groups, the risk difference was 11.9% (95% CI: 3.3%–20.6%; *p* = 0.017). After Bonferroni correction, the adjusted *p* value was 0.05, which exceeded the corrected significance threshold of 0.0167 and was therefore not considered statistically significant (Table 3). No significant differences were observed between the povidone‐iodine and control groups or between the povidone‐iodine and metronidazole gel groups after Bonferroni correction (Table 3).

#### 3.2.3 Surgical Site Infection

Although a decreasing trend was observed from 11/67 (16.42%) in the control group to 7/67 (10.45%) in the povidone‐iodine group and 4/67 (5.97%) in the metronidazole gel group, the overall difference among the three groups was not statistically significant (chi‐square test, *p* = 0.15) (Table 2).

#### 3.2.4 Composite Infectious Morbidity (SSI + Fever + Endometritis)

The composite outcome occurred in 21/67 (31.34%) in the control group, 10/67 (14.93%) in the povidone‐iodine group, and 6/67 (8.96%) in the metronidazole gel group. In pairwise comparisons (Table 3), metronidazole gel significantly reduced the composite outcome compared with the control group (risk difference: 22.4%; 95% CI: 9.3%–35.4%; *p* = 0.001; Bonferroni‐adjusted *p* = 0.003). Povidone‐iodine also reduced the composite outcome compared with the control group (risk difference: 16.4%; 95% CI: 2.4%–30.4%; *p* = 0.02; Bonferroni‐adjusted *p* = 0.07), but this difference did not reach statistical significance after adjustment. No significant difference was observed between metronidazole gel and povidone‐iodine (risk difference: 6.0%; 95% CI: −4.9% to 16.9%; *p* = 0.29; Bonferroni‐adjusted *p* = 0.86).

### 3.3 Secondary Outcome

#### 3.3.1 Need for Additional Antibiotics

The need for additional antibiotics was 11/67 (16.42%) in the povidone‐iodine group, 7/67 (10.45%) in the metronidazole gel group, and 18/67 (26.87%) in the control group (chi‐square test, *p* = 0.04) (Table 2). In the pairwise comparison between the control and metronidazole groups, the risk difference was 16.4% (95% CI: 3.5%–29.3%; *p* = 0.015). However, after Bonferroni correction, the adjusted *p* value was 0.05, which exceeded the corrected significance threshold of 0.0167 and was therefore not considered statistically significant (Table 3). No significant differences were observed between the povidone‐iodine and control groups or between the povidone‐iodine and metronidazole gel groups after Bonferroni correction (Table 3).

#### 3.3.2 Readmission and Length Of Hospital Stay

No statistically significant differences were observed among the groups for readmission (chi‐square test, *p* = 0.60) or length of hospital stay (chi‐square test, *p* = 0.78) (Table 2).

### 3.4 Multivariable Analysis

To adjust for potential confounders, a multivariable logistic regression model was performed for the composite infectious morbidity outcome. After adjustment for age, BMI, gestational age, preoperative hemoglobin, gravidity, parity, and education level, metronidazole gel remained independently associated with a significantly lower odds of composite infectious morbidity compared with the control group (adjusted OR: 0.21; 95% CI: 0.08–0.58; *p* = 0.002). Povidone‐iodine also showed a significant protective effect compared with the control group (adjusted OR: 0.38; 95% CI: 0.16–0.89; *p* = 0.03) (Table 4). None of the other covariates were statistically significant predictors of the composite outcome.

**Table 4:** Multivariable logistic regression analysis for the composite outcome.

Variable	Adjusted odds ratio (OR)	95% Confidence interval	*p* value
Group (reference: Control)			
Povidone‐iodine	0.38	(0.16–0.89)	0.03
Metronidazole gel	0.21	(0.08–0.58)	0.002
Age (years)	1.02	(0.96–1.08)	0.51
BMI (kg/m^2^)	1.04	(0.94–1.14)	0.41
Gestational age (weeks)	0.92	(0.47–1.79)	0.80
Preoperative Hb (g/dL)	0.85	(0.58–1.24)	0.40
Gravidity	1.18	(0.87–1.60)	0.28
Parity	1.10	(0.74–1.64)	0.64
Education level (reference: Primary)			
Secondary education	0.92	(0.45–1.88)	0.82
Tertiary education	0.78	(0.23–2.61)	0.68

*Note:* Dependent variable: Composite outcome (including endometritis, fever, and surgical site infection). Statistical significance was set at *p* < 0.05.

### 3.5 Adverse Events

No serious adverse events were reported in any of the three groups. Mild, transient local discomfort was reported in two patients (3.0%) in the povidone‐iodine group; these resolved spontaneously without intervention. No adverse events were reported in the metronidazole gel or control groups.

## 4 Discussion

To the best of our knowledge, this is the first randomized controlled trial directly comparing the efficacy of 0.75% metronidazole gel with 10% povidone‐iodine solution for vaginal preparation before cesarean delivery. The main finding of this study was that the use of metronidazole gel before surgical incision significantly reduced the composite infectious outcome (8.96% vs. 31.34%; risk difference: 22.4%; 95% CI: 9.3%–35.4%; Bonferroni‐adjusted *p* = 0.003). The reductions in endometritis, fever, and the need for additional antibiotics in the metronidazole group did not remain statistically significant after adjustment for multiple comparisons; although the individual components did not remain statistically significant after adjustment, the consistent direction of the observed effects suggests potential clinical relevance, which warrants further investigation in larger, adequately powered studies. This finding is important for two reasons. First, from a public health perspective, each course of additional antibiotics carries the risk of drug‐related adverse effects and, more importantly, contributes to the growing threat of antimicrobial resistance [20]. Second, from an economic perspective, a reduced need for additional antibiotics translates into lower treatment costs for both patients and the healthcare system [21]. Although a formal cost‐effectiveness analysis was beyond the scope of this study, povidone‐iodine solution is generally considered less expensive than metronidazole gel. However, the lower rate of additional antibiotic use observed in the metronidazole group suggests that its higher acquisition cost may be partially offset by reduced postoperative treatment requirements. Future studies incorporating formal economic evaluations are warranted. In contrast, although the efficacy of the povidone‐iodine group did not reach statistical significance in the unadjusted pairwise comparisons after Bonferroni correction, multivariable logistic regression analysis confirmed that both povidone‐iodine (adjusted OR: 0.38, 95% CI: 0.16–0.89; *p* = 0.03) and metronidazole gel (adjusted OR: 0.21, 95% CI: 0.08–0.58; *p* = 0.002) were independently associated with lower odds of composite infectious morbidity compared with the control group. These findings suggest that the observed treatment effects were not attributable to baseline differences among participants and support the robustness of the primary results.

### 4.1 Explaining the High Baseline Infection Rate: Intersection of Individual and Environmental Risk Factors

The baseline rates of endometritis (11.94%) and SSI (16.42%) in our control group were considerably higher than those reported in developed countries (e.g., endometritis: 0.7% in the UK and 7.2% in the US) [22, 23]. In contrast, our findings are consistent with studies from other resource‐limited settings, where SSI rates of 19.7% in Ethiopia, 18.5% in Nigeria, and 33.3% in Egypt, and endometritis rates of 29.6% in Egypt and 9.2% in Nigeria have been reported [3, 24, 25]. These figures also align with the findings of a large global meta‐analysis by Islam et al. (2025) involving 271,954 women, which showed that SSI rates in low‐middle‐income countries (8.0%) were significantly higher than in high‐income countries (5.0%) [26].

The high baseline infection rate can be explained by a convergence of individual and environmental risk factors. **First**, obesity (BMI ≥ 30) is one of the strongest independent risk factors for post‐cesarean infection. A meta‐analysis by Guo et al. (2025) identified obesity as the strongest predictor of SSI (OR = 3.0) [27]. The mean BMI in our population was 30.4 (class I obesity). Moreover, most of our patients were of low socioeconomic status, with 48.8% having less than a high school education; a systematic review by Malmire et al. (2022) identified low education level as a key factor in postpartum infection [28]. In addition, the mean gravidity in our population was 2.7, and multiple studies have identified multiparity as an independent risk factor for post‐cesarean infection [29]. **Second**, the mean preoperative hemoglobin level was 11.8 g/dL (mild anemia) and postoperative hemoglobin was 10.6 g/dL. Low hemoglobin impairs the immune system and tissue repair; Olsen et al. (2010) found that anemia was associated with a 2.18‐fold increase in endometritis (OR = 2.18, 95% CI: 1.30–3.68) [30]. **Third**, this trial was conducted at a busy, high‐volume teaching referral center. The presence of residents and medical students in the surgical team prolongs operative time and increases tissue manipulation, both of which are associated with a higher risk of infection, as confirmed by Shree and Shacho [25, 31]. In summary, the intersection of these factors provides a scientific explanation for the high baseline infection rate in the control group and highlights why topical preventive interventions are crucial in such settings.

### 4.2 Possible Explanations for the More Favorable Outcomes With Metronidazole Gel Compared With Povidone‐Iodine

Although both vaginal preparation methods were intended to reduce the microbial load, our clinical findings showed that metronidazole gel was associated with lower rates of infectious morbidity than povidone‐iodine solution. Because no microbiological assessments were performed in the present study, the reasons for this observed difference cannot be directly determined from our data. However, several biological mechanisms proposed in the literature may help explain the findings.

#### 4.2.1 First: Physical Barrier Dynamics Versus Transient Contact

Unlike aqueous solutions, the gel formulation has bioadhesive properties that may allow it to adhere to the vaginal mucosa [32]. A landmark study by Pitt et al. (2001) demonstrated that systemic absorption of metronidazole gel is less than 2% of an oral dose [33]. This local retention could enable the gel to remain in the vaginal fornices throughout surgery (30–60 min), potentially acting as a physicochemical barrier that prevents bacterial ascent. In contrast, povidone‐iodine is merely a transient irrigating solution that is rapidly diluted and washed away by amniotic fluid and blood during surgery, closing its protective window [34].

#### 4.2.2 Second: Targeted Therapy Versus Ecological Dysbiosis

From a microbiological perspective, metronidazole is a highly targeted antibiotic that may selectively inhibit anaerobic pathogens while causing minimal harm to lactobacilli (the protective vaginal flora) [35]. In contrast, povidone‐iodine is a broad‐spectrum antiseptic that may destroy microorganisms indiscriminately [36]. Consequently, using 10% povidone‐iodine may create a microbial vacuum and disrupt the natural protective flora, a situation that could exacerbate dysbiosis and facilitate colonization by opportunistic pathogens.

#### 4.2.3 Third: Tissue Toxicity and Local Immune Impairment (Mucosal Cytotoxicity)

Although 10% povidone‐iodine has strong bactericidal properties, it may be toxic and irritating to fibroblasts and vaginal epithelial cells [37]. Damage to the integrity of the vaginal mucosa could paradoxically weaken the natural local immune barrier and facilitate bacterial entry. This argument is consistent with the findings of a meta‐analysis by Liu et al. (2023), which showed that lower concentrations (1%) of povidone‐iodine are more effective than higher concentrations (10%), because higher concentrations may exert locally damaging effects [38]. In contrast, the water‐based formulation of metronidazole gel is not only non‐toxic but also well tolerated by the vaginal mucosa [39].

Taken together, these proposed mechanisms remain speculative because no microbiological measurements were performed in the present study. Future studies incorporating vaginal microbiological assessments are needed to determine whether these hypotheses explain the observed clinical findings.

### 4.3 Local Epidemiology of Bacterial Vaginosis and Differential Pathogenesis of Infections

A possible explanation for the favorable outcomes observed with metronidazole in reducing endometritis requires consideration of the local epidemiology of bacterial vaginosis (BV). The prevalence of BV in the Iranian pregnant population is estimated to be approximately 36% [40], which is considerably higher than the global average (23%–29%) [41]. Pregnancy, particularly in the third trimester, is associated with a decrease in protective lactobacilli, shifting the balance in favor of anaerobic overgrowth [42]. Classic studies have shown that BV is a very strong risk factor for post‐cesarean endometritis (OR = 5.8, 95% CI: 3.0–10.9) [43]. Metronidazole is recommended as first‐line therapy for BV, and multiple clinical trials have confirmed its efficacy in eradicating anaerobic flora [44]. Therefore, it is plausible that reducing the vaginal anaerobic burden before cesarean delivery may contribute to a lower risk of endometritis, although this mechanism was not directly evaluated in the present study.

#### 4.3.1 Explaining the Lack of a Significant Reduction in Surgical Site Infection (SSI)

Metronidazole gel produced a marked reduction in endometritis (from 11.94% to 1.49%) and a considerable reduction in SSI (from 16.42% to 5.97%), but the reduction in endometritis was much more pronounced than that in SSI, which can be explained by the differential pathogenesis of these two infections. Endometritis is an ascending infection originating from the vaginal anaerobic flora [6, 7], whereas SSI is predominantly exogenous, caused by aerobic bacteria from the skin flora, especially *Staphylococcus aureus* [45]. Consequently, reducing the vaginal microbial load does not directly affect the skin flora. This pattern was also observed in a study by Pitt et al. (2001), in which metronidazole gel reduced the endometritis rate from 17% to 7% but showed no significant difference in wound infection rates between groups [33]. Even in studies where povidone‐iodine achieved a significant reduction in endometritis, this benefit did not extend to wound infection [24]. These findings are consistent with a meta‐analysis by Lawerd et al. (2025), which showed that vaginal preparation has a smaller effect on SSI than on endometritis [12]. Taken together, these observations emphasize that vaginal interventions are much more effective in reducing endometritis (endogenous origin) than SSI (exogenous origin), and this pattern appears to be independent of the specific antiseptic agent used.

### 4.4 Explaining the Apparent Contradictions: The “Baseline Risk” Paradigm

In apparent contrast to our findings, several studies from developed countries have shown no benefit of vaginal preparation. Reid et al. (2001) in the US, studying 498 women, found no benefit of vaginal cleansing with Betadine [23]. Temming et al. (2023), in a multicenter US trial of 608 women, reported that vaginal washing with povidone‐iodine before unscheduled cesarean delivery did not reduce the composite infectious outcome (11.8% vs. 11.5%; *p* = 0.90) [6]. Similarly, the PREPS trial in the UK found that vaginal preparation with chlorhexidine was ineffective [22].

All these apparent contradictions can be resolved by a fundamental epidemiologic principle: **the difference in baseline infection risk**. In populations where the baseline infection risk is already very low (e.g., developed countries), the effect of topical preventive interventions is masked and appears statistically futile. In contrast, in our high‐risk population, these interventions appeared to be associated with greater reductions in infectious morbidity. This reasoning is supported by a subgroup analysis from the meta‐analysis by Liu (2023), which demonstrated that the protective effect of vaginal preparation is much more pronounced and critical in developing countries [38]. Therefore, local baseline risk and regional epidemiology should be considered when applying evidence from other healthcare settings, and further studies are needed before broad recommendations can be made.

## 5 Limitations

Despite its methodological and clinical strengths, this study has several limitations. First, blinding of the intervention provider and outcome assessor was not feasible due to the different physical nature of the interventions. The lack of blinding of intervention providers may have introduced performance bias; however, the standardized surgical protocol and prophylactic antibiotic administration were applied equally to all groups, minimizing this risk. The lack of blinding of outcome assessors may have introduced detection bias; however, objective criteria (thermometry, purulent discharge) and standardized CDC/NHSN definitions were used to minimize this bias. Second, the sample size calculation was based on endometritis because it was the least frequent yet clinically most important infectious outcome. We assumed a relatively large effect size based on a previous study conducted in a similar high‐risk setting. We acknowledge that this assumption may have been somewhat optimistic and may have reduced the statistical power to detect differences for less frequent outcomes. Nevertheless, the composite infectious morbidity outcome, which included endometritis, surgical site infection, and postoperative fever, occurred more frequently than endometritis alone and was therefore unlikely to have had lower statistical power than the outcome used for the original sample size calculation. Although the composite outcome remained statistically significant after adjustment for multiple comparisons, some pairwise comparisons approached the threshold of statistical significance, suggesting that the study may have been underpowered for certain secondary outcomes. Third, only elective cesarean deliveries were included, so generalization to unscheduled cesarean deliveries requires further studies. Fourth, the lack of vaginal microbiological cultures precluded definitive proof of the mechanism of microbial load reduction. Finally, this was a single‐center study conducted in a tertiary referral hospital with a relatively high baseline risk of post‐cesarean infection. Therefore, local epidemiological characteristics may limit the generalizability of these findings to lower‐risk populations. Future multicenter studies are warranted.

## 6 Conclusion

This trial provides robust evidence that preoperative vaginal preparation with 0.75% metronidazole gel significantly reduced the composite infectious outcome in high‐risk populations with a high baseline infection rate. Although the individual components (endometritis, postoperative fever, and need for additional antibiotics) showed clinically meaningful reductions, they did not reach statistical significance after adjustment for multiple comparisons. Due to its tissue retention and selective targeting of anaerobes without harming the vaginal ecosystem, metronidazole gel may represent a promising alternative to 10% povidone‐iodine.

Metronidazole gel also offers a safe alternative for patients with iodine sensitivity or thyroid disorders. These findings fill an important gap in the international evidence base and recommend that instead of relying exclusively on guidelines from developed countries, preventive interventions should be tailored to local baseline risk and regional infection epidemiology.

## Author Contributions

**Fatemeh Davodabadi:** project administration, formal analysis, writing – original draft, visualization. **Hamideh parsapour:** validation, project administration, methodology. **Behzad Imani:** Writing – review and editing, supervision. **Salman Khazaei:** software, validation, formal analysis. **Mahnaz Moghadari Koosha:** investigation, software.

## Ethics Statement

This study was reviewed and approved by Ethics Committee of Hamedan University of Medical Sciences with the approval number: IR.UMSHA.REC.1404.821.

## Conflicts of Interest

The authors declare no conflicts of interest.

## Transparency Statement

Behzad Imani affirms that this manuscript is an honest, accurate, and transparent account of the study being reported; that no important aspects of the study have been omitted; and that any discrepancies from the study as planned (and, if relevant, registered) have been explained.

## Data Availability

Data associated with the study has not been deposited into a publicly available repository. Data are available from the corresponding author on reasonable request.
